# Determination and Prediction of the Net Energy Content of Barley for Gestating Sows

**DOI:** 10.3390/ani16071095

**Published:** 2026-04-02

**Authors:** Zirou Yu, Zongliang Li, Tong Hu, Tingting Li, Fenglai Wang, Hu Liu

**Affiliations:** 1 State Key Laboratory of Animal Nutrition, College of Animal Science and Technology, China Agricultural University, Beijing 100193, China; yuzirou0828@163.com (Z.Y.); 15195931240@163.com (T.H.); wangfl@cau.edu.cn (F.W.); 2 China Animal Husbandry Nutrition (Beijing) Technology Co., Ltd., Beijing 100071, China; 18253684733@163.com; 3 College of Veterinary Medicine, China Agricultural University, No. 2 Yuanmingyuan West Road, Beijing 100193, China; ltt@cau.edu.cn

**Keywords:** barley, gestating sows, indirect calorimetry, net energy

## Abstract

This study determined the net energy (NE) value of barley for gestating sows and established net energy prediction equations by using indirect calorimetry. Ten barleys were selected for net energy determination in gestating sows and equation development. This experiment adopted a 4 × 6 × 3 Youden square design with 11 treatments: 1 corn-soybean basal diet and 10 barley diets containing 28.9% barley. Twenty-four healthy multiparous sows at 30–40 d of gestation were allocated and measured digestible energy, metabolizable energy and fasting heat production. Results showed that the digestible energy and metabolizable energy values of the barley ranged from 12.08 to 17.28 MJ/kg DM and 11.74 to 15.39 MJ/kg DM, with a mean value of 15.02 MJ/kg DM and 13.88 MJ/kg DM. By performing stepwise regression analysis of the net energy values of these ten barley ingredients and their chemical compositions, the prediction equation for the net energy of barley fed to gestating sows was obtained as NE = 4.35 − 3.92 ADF + 1.24 TDF (R^2^ = 0.66, *p* = 0.01). When metabolizable energy was included in the equation, it was obtained as NE = 10.21 + 0.93 ME + 0.51 CP (R^2^ = 0.62, *p* = 0.03).

## Simple Summary

Barley is widely available and has the potential to replace part of the corn used in pig diets, but information on its energy value for gestating sows is still limited. In this study, ten barley samples were tested in 24 gestating sows by indirect calorimetry. The results showed wide variation in the digestible, metabolizable, and net energy values of barley, suggesting that different barley sources do not provide the same feeding value. Chemical characteristics, fiber-related fractions and crude protein were associated with this variation and were useful for predicting net energy. This study provides practical data for improving the nutritional evaluation of barley for gestating sows and may help nutritionists formulate diets more precisely.

## 1. Introduction

In the current energy feed systems for pigs, corn and wheat serve as the primary energy sources, with corn being particularly prominent [1]. In contrast, commercial utilization of unconventional resources such as barley remains underdeveloped. Structural imbalances within global feed ingredient supply chains have exacerbated volatility in the availability and pricing of bulk commodities like corn and soybeans, directly elevating feed production costs. To mitigate over-dependence on imported corn and soybean meal, strategic transformation through diversified feed formulation systems and innovative technological pathways has become imperative.

Barley, as a principal cereal grain, represents an energy-dense feed ingredient with substantial nutritional value comparable to corn. It is notably rich in insoluble dietary fiber (IDF), including anti-nutritional factors such as non-starch polysaccharides, which consequently reduces its gross energy (GE) relative to corn. Dietary fibers are categorized by analytical methodology into four types: crude fiber, dietary fiber, acid detergent fiber (ADF), and neutral detergent fiber (NDF) [2]. The limited digestibility of IDF and associated components in pigs diminishes barley’s feeding value. When incorporated at elevated inclusion rates in swine diets, pretreatment or enzymatic supplementation becomes necessary. Despite these limitations, barley offers significant advantages, including lower susceptibility to fungal contamination, enhanced palatability, and cost-effectiveness, positioning it as a promising unconventional feed resource. Despite increasing research on barley in recent years, the net energy (NE) values for barley in NRC (2012) [3] remain predominantly derived from growing pigs [4]. Given the developmental stage-dependent nutrient utilization in swine, NE data specific to gestating sows fed barley diets remain notably scarce.

To promote the application of barley as an unconventional feed ingredient in pig production, the current energy evaluation systems to estimate feed components for swine have evolved from the Digestible Energy (DE) system to the Metabolizable Energy (ME) system, and subsequently to the NE system. However, both the DE and ME systems underestimate the energy value of fat and starch components [5] and overestimate the energy value of protein and fiber components [6], which suggests limitations in predicting the energy value of barley for pigs. In contrast, the NE system accounts for the heat increment (HI) [4] and provides more accurate estimations of the energy value for fiber-rich feedstock than DE or ME systems. The NE system offers advantages in evaluating swine feed ingredients and better predicts growth and production performance [7]. This study will focus on gestating sows to determine the effective energy values of barley and establish NE prediction equations.

## 2. Materials and Methods

### 2.1. Animals, Diets and Experimental Design

This experiment was conducted at the Fengning Animal Experimental Base of China Agricultural University (Hebei Province, China). The experimental procedures completed in the study were approved by the Animal Care and Use Committee of China Agricultural University (AW71802202-1-1, Beijing). To ensure that the selected barley samples are from different origins and have significant variances in chemical composition, a total of seven domestically produced barley and four imported barley samples were eventually determined (Table 1). Twenty-four multiparous sows (Yorkshire × Landrace; 2 to 5 parity) at 30~40 d of gestation with an initial body weight (BW) of 213 ± 17.8 kg were selected.

The experiment employed a 4 × 6 × 3 Yuden design. Based on the nutritional levels of 10 different barley varieties, the samples were divided into two batches for experimental analysis. Each Yuden design included six treatment groups: one control diet group and five barley diet groups, which were formulated by replacing 28.9% of the corn and soybean meal in the control diet with different barley ingredients.

**Figure 1:**
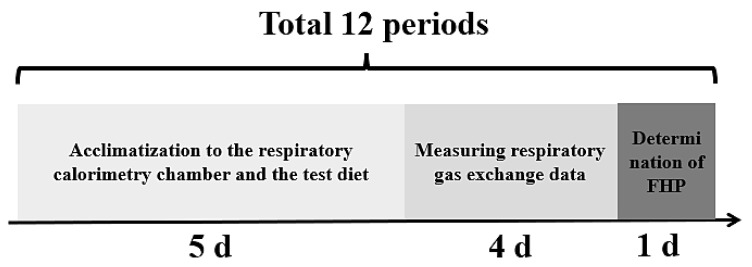
Schematic representation of the 10 d experimental period for gestating sows.

A schematic overview of each 10 d experimental period is shown in Figure 1, including the 5 d acclimatization period, 4 d of respiratory gas exchange measurement, and 1 d for the determination of fasting heat production (FHP). During the acclimatization period, sows in metabolic cages were transferred to an open respiratory calorimetry chamber to acclimatize to the metabolic cage and respiratory chamber environments. Respiratory gas exchange was measured on d 6 through d 8, and FHP was measured on d 9. During the calorimetry period, O_2_, CO_2_ and CH_4_ gas concentrations data were measured with the total collection of feces and urine. On d 9, sows were fasted, and only urine was collected. Fasting heat production was calculated using gas exchange data from 22:00 (d 9) to 06:00 (d 10). In each period, sows were weighed at the beginning of the experimental period (d 5) and at the end of d 10.

Sows were fed 1.3 times (544 kJ ME/kg BW^0.75^ d^−1^) the maintenance requirement of gestating sows daily. Diets were fed in equal portions at 8:30 and 15:30 daily. The ambient temperature of the chambers was maintained at 20 ± 1 °C, and the relative humidity at about 70%. Lights remained on from 7:00 to 19:00, daily. The chemical composition and nutrient content of the barley and experimental diets are presented in Table 1 and Table 2.

### 2.2. Sample Analysis and Calculation

Ingredients, diets, and fecal samples were analyzed for DM (method 930.15), crude protein (CP, method 984.13), and ash (method 942.05) concentration [8]. Starch content of ingredients and diets was measured by the enzymatic-colorimetric method [9]. The ether extract concentration of ingredients and diets was analyzed according to the procedure described by Thiex et al. [10]. Total dietary fiber, soluble dietary fiber, and insoluble dietary fiber of ingredients and diets were determined using method 991.43 [8]. Concentration of 18 amino acids of ingredients and diets was determined according to method 982.30 E [8] using High Performance Liquid Chromatograph (HPLC, Agilent 1200 series, Agilent Technologies, Santa Clara, CA, USA) and an amino acid analyzer (Hitachi L-8900, Hitachi, Tokyo, Japan). Neutral detergent fiber and ADF of ingredients, diets, and fecal samples were determined according to the procedure of Vansoest et al. [2]. GE content of ingredients, diets, urine, and fecal samples was analyzed using an isoperibol calorimeter (Parr 6300 Calorimeter, Parr Instrument Company, Moline, IL, USA) according to the method of international standard ISO9831:1998 [11].

Oxygen concentration was determined using a paramagnetic oxygen analyzer (Oxymat6E, Siemens, Munich, Germany), CO_2_ and CH_4_ concentrations were determined using an infrared analyzer (Ultramat6E, Siemens, Germany), and respiratory chamber exhaust volume was determined using a mass flow meter (Alicat, Tucson, AZ, USA). Total heat production (THP) was calculated as described by Brouwer [12]: THP (kJ) = 16.18 × O_2_ (L) + 5.02 × CO_2_ (L) − 2.17 × CH_4_ (L) − 5.99 × urinary nitrogen excretion (g). Calculation of retained dietary energy (RE) and partitioning of RE were conducted according to the equation described by Labussiere et al. [13]. The apparent total tract digestibility of nutrients in wheat bran was calculated according to the procedure of He et al. [1].

### 2.3. Statistical Analysis

In the present study, each sow was considered as an experimental unit. Experimental data were analyzed by one-way ANOVA using the GLIMMIX model in SAS statistical software (version 9.4, SAS Institute Inc., Cary, NC, USA). Tukey’s multiple comparisons were used, *p* < 0.05 was considered statistically significant difference, and 0.05 ≤ *p* ≤ 0.1 were considered statistical trends. The relationship between nutritional value and chemical composition was determined using Proc CORR. Stepwise regression was performed using the Proc REG program to establish a prediction equation for the net energy content of barley. The coefficient of determination (R^2^), root-mean-square error (RMSE) and *p*-value were used as indicators to evaluate the best prediction equation. The statistically significant equation with the largest coefficient of determination (R^2^) and the smallest RMSE was considered the best prediction equation.

## 3. Results

### 3.1. Effects of Barley Diets on Nutrient Digestibility, Nitrogen Balance and Energy Utilization of Gestating Sows

Digestibility of DM and OM of the basal diet was higher (*p* < 0.01) than in the BL-2~BL-9 diets, and GE digestibility was significantly higher than that of the BL-2, BL-6~BL-9 diets (*p* < 0.01). CP, ADF and EE digestibility of barley diets were different (*p* < 0.05). Among the barley-containing diets, BL-1 showed the highest digestibility values for GE, DM, CP, OM, and EE. There was a significant difference in the ingested nitrogen and urinary excretion of nitrogen among barley diets (*p* < 0.01). Urinary excretion of nitrogen was higher in BL-6 (*p* < 0.01), but there were no significant differences in fecal excretion of nitrogen or nitrogen deposition. There were no significant differences in UE/DE, CH4/DE, ME/DE and NE/ME among the experiment diets (Table 3).

### 3.2. Effects of Barley Diets on Energy Balance of Gestating Sows

The energy balance variables of the complete experimental diets are presented in Table 4. No significant differences were observed among treatments in ME intake, THP, FHP, or retained energy as protein (REP). ME intake ranged from 507 to 531 kJ/kg BW^0.75^/d among the barley diets, while THP ranged from 408 to 476 kJ/kg BW^0.75^/d and FHP ranged from 349 to 380 kJ/kg BW^0.75^/d. Retained energy as protein varied numerically from 55 to 81 kJ/kg BW^0.75^/d, but these differences were not statistically significant.

In contrast, retained energy as lipid (REL) and total retained energy (RE) differed significantly among treatments. The lowest REL value was observed in BL-5, whereas the highest values were observed in BL-7 and BL-10, indicating marked variation in lipid energy retention among diets. Total retained energy ranged from 29 to 138 kJ/kg BW^0.75^/d, with BL-7 showing the greatest RE among the barley diets. Respiratory quotient also differed significantly among treatments in both the fed and fasted states. In the fed state, RQ ranged from 0.93 to 1.00, whereas in the fasted state it ranged from 0.76 to 0.83. Digestible energy of the complete diets also differed significantly, ranging from 14.50 to 15.88 MJ/kg DM among the barley diets, with the highest DE value observed in BL-4. However, no significant differences were detected in dietary ME or NE values among treatments (Table 4).

### 3.3. Nutrient Digestibility and Energy Value of Barley

There were no significant differences in DM, GE, OM, NDF and CP digestibility among the different barleys. Digestibility of ADF for BL-4 was significantly higher than that of the other eight barleys except BL-1 diets (*p* < 0.05). DE value was different (*p* < 0.05) among the 10 barleys, but ME, NE and energy conversion efficiencies were not significantly different. The average efficiency of conversion of DE to ME and ME to NE of barley was 92.40% and 70.4%. DE, ME, and NE of the 10 barleys averaged 15.02, 13.88, and 9.96 MJ/kg DM, respectively. NE for the seven domestic barley varieties evaluated were 8.88, 9.66, 11.61, 8.49, 11.18, 10.95, and 11.85 MJ/kg DM, and NE for three imported barley ingredients were 9.22, 8.49 and 9.95 MJ/kg DM (Table 5).

### 3.4. Correlation Analysis and Net Energy Prediction Equations for Gestating Sows

The correlation coefficients among the chemical composition and energy values of the ten barley samples are shown in Table 6. Both ME and NE were positively correlated with DE (*p* < 0.05), and NE was also positively correlated with ME (*p* < 0.05). Digestible energy was negatively correlated with CP and ADF content, whereas ME was negatively correlated with EE content (*p* < 0.05). In addition, CP was positively correlated with GE, IDF, and Ash content (*p* < 0.05). Among the fiber-related variables, hemicellulose was very strongly positively correlated with NDF (R^2^ = 0.99, *p* < 0.01), because hemicellulose is an important component of the NDF fraction. Likewise, IDF was very strongly positively correlated with TDF (R^2^ = 0.97, *p* < 0.01), indicating that total dietary fiber in the barley samples was mainly contributed by the insoluble fraction. ADF was also strongly positively correlated with IDF (R^2^ = 0.94, *p* < 0.01) and TDF (R^2^ = 0.92, *p* < 0.01), suggesting that variation in total fiber among barley samples was largely associated with variation in insoluble structural fiber. In contrast, starch was negatively correlated with NDF, hemicellulose, and Ash, reflecting the compositional trade-off between structural carbohydrates and starch in barley. The predictive equation for NE was NE = 4.35 − 3.92 ADF + 1.24 TDF (R^2^ = 0.66, *p* = 0.01). When metabolizable energy is included in the equation, it is obtained as: NE = 10.21 + 0.93 ME + 0.51 CP (R^2^ = 0.62, *p* = 0.03) (Table 7).

## 4. Discussion

### 4.1. Differences in Chemical Composition of Barley

Previous studies have indicated that the variability of barley is mainly associated with fiber-related fractions, especially NDF, ADF, and ash content [2]. The results of the present study are consistent with this general pattern. Among the ten barley samples evaluated, CP ranged from 9.29% to 14.26% DM, NDF ranged from 21.79% to 39.54% DM, ADF ranged from 3.65% to 6.07% DM, and ash ranged from 2.10% to 3.62% DM, indicating substantial compositional heterogeneity among samples. In particular, the variation in NDF was pronounced, which suggests that differences in structural carbohydrate content were one of the major sources of variation among barleys. Compared with tabulated values reported by NRC [3], the NDF values measured in the present study were generally higher. This difference may partly explain the lower digestibility and energy values observed for some barley samples, because elevated fiber content is known to dilute the starch fraction and reduce the digestible energy contribution of cereal grains. In addition, the ranges of variation for CP and ash in the present study were broader than those reported by Fairbairn et al. [14], who reported CP values of 11.8–15.1% DM and ash values of 2.8–3.2% DM. This comparison further confirms that the barley samples used in the present experiment exhibited considerable chemical diversity. The barley samples were intentionally selected from different origins in order to maximize variation in chemical composition. Therefore, differences in cultivar, growing environment, and post-harvest conditions may all have contributed to the observed heterogeneity.

### 4.2. Effects of Barley Diets on Nutrient Digestibility, Nitrogen Balance and Energy Balance of Gestating Sows

The addition of barley reduced the digestibility of dietary nutrients, which aligns with previous findings [15]. The tight integration of lemma hulls and kernels in hulled barley makes them difficult to separate, resulting in a high content of barley hulls in feeding barley. This increases fiber components in the raw material and reduces the digestibility of nutrients. The digestibility of GE, DM, OM, CP, and EE in the experimental diets was consistent with previous studies [16,17,18], while NDF and ADF digestibility remained relatively low. The reason for the difference may have been the greater body weight of sows, as well as lower barley inclusion ratio, and reduced dietary fiber content. Furthermore, the lower NDF digestibility could also stem from incompletely grinding barley hulls, leading to incomplete fiber digestion in the sows. This observation broadly corroborates reports that fiber reduces the digestibility of other nutrients [19]. Compared to the basal diet, the apparent CP digestibility of the 10 barley-containing mixed diets decreased, consistent with the results of nitrogen loss from feces. The digestion of barley fiber in swine elevates endogenous losses, primarily comprising protein-rich epithelial cells, mucus, and digestive enzymes. Additionally, enhanced microbial protein anabolism may contribute to this phenomenon, both mechanisms leading to increased fecal nitrogen loss and ultimately reduced CP digestibility [20]. Grinding barley to medium fineness improves its feeding value compared to whole-grain feeding. However, excessive grinding may increase the risk of gastrointestinal ulcers in animals [21]. Barley that has been extruded also exhibits improved nutrient digestibility. These findings suggest that appropriate processing of barley diets may enhance nutrient digestibility in gestating sows, though further research is required.

The average total heat production of the barley diets was consistent with previous studies [15], whereas fasting heat production showed elevated values. Research indicates that fasting heat production increases with gestational days [17]. Due to farm constraints, the sows selected for this trial were at around 40 d of gestation, with the later experimental period approaching late gestation. When extrapolated to fasting heat production of gestational 0 d through regression analysis, the results closely aligned with those reported by Le Goff and Noblet [22], suggesting gestational progression as the primary factor causing fasting heat production variations. During late experimental stages, increased heat production accompanied by reduced RQ was observed in some sows, consistent with findings of Young et al. [23]. Analysis revealed that dietary NDF content and total heat production during feeding were correlated positively. Variations in barley fiber intake led to differences in chewing duration and intestinal fermentation efficiency as well as peristaltic activity, which ultimately alter heat production. Compared to early and mid-gestation, late-gestation sows demonstrated greater heat production, lower energy retention, and diminished lipid deposition [16].

Significant differences were observed in the RQ between the fed state and the fasting-state RQ in all treatment groups. The RQ values of BL-4, BL-5, and BL-9 were close to those reported by Le Goff et al. [18], while the RQ values of other groups were below 1. The decrease in RQ was associated with lower ME intake in sows. Although the feeding level was designed at 1.3 times maintenance requirements, the actual ME intake of sows remained suboptimal, which may explain the lower RQ. The conversion efficiency of dietary DE to ME was 92.72%, slightly lower than the value reported by Le Goff et al. [18] for adult sows, likely due to excessive urinary nitrogen loss. The higher fecal and urinary nitrogen losses in barley-based diets may be attributed to fiber components accelerating gastrointestinal motility and increasing endogenous nitrogen excretion [19]. Nitrogen intake level showed no correlation with fecal or urinary nitrogen losses, as variations in body weight led to differences in feeding amounts, thereby affecting ME intake and nitrogen levels. Compared to growing pigs, gestating sows fed barley exhibited higher nitrogen losses.

### 4.3. Effect of Barley on Energy Balance

The average DE and ME of the ten barleys were lower than the values reported by Ramonet et al. and Le Goff et al. [17,18]. Some studies show that dietary fiber level and fiber source markedly influence nutrient digestibility and energy utilization in pigs and sows [17,18,19]. Fiber-rich ingredients can reduce digestibility and modify heat production and energy partitioning, which is consistent with the variation observed among the barley samples evaluated here. The average NE of the experimental diets was 9.96 MJ/kg DM, lower than findings for barley in growing pigs [24]. This discrepancy may result from high barley inclusion reducing nutrient digestibility and subsequently energy value. The strong correlation between NE and DE/ME values in this study suggests stable conversion efficiency from DE to ME and NE, supporting the prediction of NE through DE and ME. However, the weak correlation between barley energy values and chemical composition implies that interactions among fiber components complicate these relationships, necessitating more precise nutrient analysis for gestating sows.

### 4.4. Effect of Chemical Compositions of Barley on the Prediction Equation of Net Energy in Gestating Sows

Net energy prediction equations were developed using ME, CP, and ADF as predictors. These factors are consistent with those in the net energy equation for grain raw materials containing barley constructed by a previous study [24], confirming that CP and fiber content are primary drivers of NE variation. By comparing NE prediction equations for barley in growing pigs, a net energy evaluation system was established for pig feed ingredients separately, one for growing-finishing pigs and another for adult sows.

Achieving precision nutrition requires determining the true nutritional value of the various feed ingredients used in the formulation, especially the CP and DF properties. Various chemical components may have different nutritional roles for different animals, and their nutritional values may vary; so, the effects of differences in barley chemical composition, especially fiber composition, on animal energy metabolism need to be further explored.

## 5. Conclusions

The differences in chemical composition of barley from different origins were mainly in CP, NDF and ADF, with coefficients of variation of 14.17%, 24.35 and 19.98%. The net energy values of 10 barleys fed to gestating sows ranged from 7.85 to 11.85 MJ/kg DM, with an average value of 9.96. The best barley net energy prediction equations obtained by stepwise regression were NE = 4.35 − 3.92 ADF + 1.24 TDF (R^2^ = 0.66, *p* = 0.01) and NE = 10.21 + 0.93 ME + 0.51 CP (R^2^ = 0.62, *p* = 0.03).

## Figures and Tables

**Table 1:** Chemical composition of ten barley samples used in gestating sows feeding experiment (%, DM basis) ^1^.

Items ^2^	Barley-1	Barley-2	Barley-3	Barley-4	Barley-5	Barley-6	Barley-7	Barley-8	Barley-9	Barley-10
DM	89.30	91.22	89.90	89.70	89.70	89.30	91.22	89.90	88.70	88.70
CP	10.87	14.07	12.32	9.29	10.84	13.06	12.98	10.88	14.26	12.62
NDF	21.79	29.38	23.16	39.54	22.48	26.75	27.38	36.59	38.36	27.87
ADF	4.30	6.07	4.79	4.73	4.62	4.80	4.31	4.92	5.93	3.65
Ash	2.15	2.63	2.78	2.59	2.48	2.54	2.73	2.10	3.62	2.47
EE	2.34	2.13	2.16	1.54	1.61	2.03	2.22	2.32	2.12	2.20
Starch	57.69	57.71	48.60	48.87	56.02	50.60	55.93	52.26	44.01	55.82
IDF	12.99	16.97	13.23	13.54	14.05	14.98	14.11	14.45	17.35	12.02
SDF	5.09	5.08	4.95	6.03	5.77	4.77	5.23	5.66	6.12	4.80
TDF	18.09	22.06	18.18	19.57	19.82	19.75	19.33	20.11	23.46	16.82
Ca	0.04	0.04	0.05	0.04	0.04	0.05	0.05	0.08	0.05	0.04
P	0.34	0.32	0.38	0.26	0.26	0.36	0.31	0.30	0.25	0.22
Indispensable AA ^3^										
Lys	0.36	0.50	0.43	0.31	0.36	0.37	0.45	0.36	0.47	0.37
Met	0.13	0.11	0.17	0.12	0.16	0.15	0.13	0.09	0.19	0.15
Thr	0.30	0.46	0.39	0.42	0.27	0.31	0.29	0.34	0.44	0.31
Val	0.47	0.70	0.64	0.61	0.39	0.50	0.44	0.50	0.65	0.50
Ile	0.32	0.47	0.44	0.43	0.25	0.34	0.32	0.35	0.45	0.36
Leu	0.60	0.97	0.85	0.84	0.53	0.65	0.63	0.66	0.93	0.68
Trp	0.14	0.17	0.17	0.16	0.11	0.16	0.14	0.14	0.17	0.15
Phe	0.56	0.87	0.64	0.69	0.40	0.51	0.57	0.53	0.80	0.72
His	0.19	0.30	0.29	0.29	0.17	0.21	0.20	0.21	0.28	0.72
Arg	0.46	0.66	0.55	0.36	0.46	0.48	0.58	0.44	0.61	0.48
Dispensable AA ^4^										
Pro	0.92	1.59	1.37	1.35	0.79	1.11	0.93	1.12	1.50	1.09
Tyr	0.33	0.54	0.25	0.33	0.22	0.24	0.34	0.25	0.46	0.49
Ser	0.36	0.57	0.49	0.50	0.31	0.39	0.35	0.41	0.53	0.39
Gly	0.36	0.55	0.47	0.47	0.33	0.38	0.38	0.40	0.53	0.38
Asp	0.53	0.70	0.69	0.71	0.51	0.47	0.53	0.60	0.68	0.51
Ala	0.37	0.56	0.50	0.52	0.35	0.39	0.38	0.40	0.52	0.40
Cys	0.15	0.20	0.21	0.19	0.16	0.19	0.20	0.16	0.24	0.19
Glu	1.87	3.09	3.04	2.80	1.56	2.10	1.76	2.19	3.06	2.10

^1^ All data are the results of a chemical analysis conducted in duplicate. ^2^ DM = dry matter; CP = crude protein; NDF = neutral detergent fiber; ADF = acid detergent fiber; EE = ethyl ether extract; IDF = insoluble dietary fiber; SDF = soluble dietary fiber; TDF = total dietary fiber; Barley-1 from Canada; Barley-2 from Anhui Province, China; Barley-3 from Gansu Province, China; Barley-4 from Yunnan Province, China; Barley-5 from Heilongjiang Province, China; Barley-6 from Kazakhstan; Barley-7 from Gansu Province, China; Barley-8 from Ukraine; Barley-9 from Henan Province, China; Barley-10 from Inner Mongolia Autonomous Region, China. ^3^ Lys = lysine; Met = methionine; Thr = threonine; Val = valine; Ile = isoleucine; Leu = leucine; Trp = tryptophan; Phe = phenylalanine; His = histidine; Arg = arginine. ^4^ Pro = proline; Tyr = tyrosine; Ser = serine; Gly = glycine; Asp = aspartic acid; Ala = alanine; Cys = cysteine; Glu = glutamic acid.

**Table 2:** Formulation and nutrient composition of experimental diets (%, as-fed basis) ^1^.

Items	Basal Diet	Barley Diet ^2^									
		BL-1	BL-2	BL-3	BL-4	BL-5	BL-6	BL-7	BL-8	BL-9	BL-10
Corn	81.43	57.18	57.18	57.18	57.18	57.18	57.18	57.18	57.18	57.18	57.18
Soybean meal	15.62	10.97	10.97	10.97	10.97	10.97	10.97	10.97	10.97	10.97	10.97
Barley-1	-	28.90	-	-	-	-	-	-	-	-	-
Barley-2	-	-	28.90	-	-	-	-	-	-	-	-
Barley-3	-	-	-	28.90	-	-	-	-	-	-	-
Barley-4	-	-	-	-	28.90	-	-	-	-	-	-
Barley-5	-	-	-	-	-	28.90	-	-	-	-	-
Barley-6	-	-	-	-	-	-	28.90	-	-	-	-
Barley-7	-	-	-	-	-	-	-	28.90	-	-	-
Barley-8	-	-	-	-	-	-	-	-	28.90	-	-
Barley-9	-	-	-	-	-	-	-	-	-	28.90	-
Barley-10	-	-	-	-	-	-	-	-	-	-	28.90
Dicalcium phosphate	1.05	1.05	1.05	1.05	1.05	1.05	1.05	1.05	1.05	1.05	1.05
Limestone	1.00	1.00	1.00	1.00	1.00	1.00	1.00	1.00	1.00	1.00	1.00
Salt	0.40	0.40	0.40	0.40	0.40	0.40	0.40	0.40	0.40	0.40	0.40
Premix ^3^	0.50	0.50	0.50	0.50	0.50	0.50	0.50	0.50	0.50	0.50	0.50
Total	100.00	100.00	100.00	100.00	100.00	100.00	100.00	100.00	100.00	100.00	100.00
Nutritional levels, DM basis											
GE kcal/kg	15.99	15.95	15.99	15.91	16.55	15.84	16.13	16.08	15.85	15.89	15.80
CP	13.74	14.10	14.06	14.78	14.20	13.83	14.35	14.83	14.34	14.26	13.99
EE	3.24	2.87	2.90	2.70	2.59	2.65	2.80	2.87	3.00	2.77	2.36
Starch	45.85	42.98	47.50	45.38	47.27	47.09	45.81	45.52	47.33	45.67	48.79
NDF	9.50	12.60	13.33	12.74	13.14	13.92	12.03	11.15	13.50	13.11	12.83
ADF	3.52	4.03	3.96	3.64	3.94	4.52	3.66	3.25	3.92	3.80	3.85
Ash	3.88	3.81	3.95	4.22	4.13	3.77	3.88	3.99	4.00	4.13	3.28

^1^ DM = dry matter; GE = gross energy; CP = crude protein; EE = ethyl ether extract; NDF = neutral detergent fiber; ADF = acid detergent fiber. ^2^ Diets BL-1~BL-10 were formulated by replacing corn and soybean meal in the basal diet with barley at a level of 28.9%. ^3^ Premix provided per kilogram of complete feed: Vitamin A, 6000 IU; Vitamin D3, 3000 IU; Vitamin E, 20 IU; Vitamin K3, 1.8 mg; Vitamin B1, 2.0 mg; Vitamin B2, 6.0 mg; Vitamin B6, 4.0 mg; Choline, 3000 mg; Vitamin B12, 0.02 mg; Niacin, 26.0 mg; Pantothenic acid, 18.0 mg; Folic acid, 3.2 mg; Biotin, 0.4 mg; Fe, 400 mg; Cu, 20 mg; Zn, 100 mg; Mn, 50 mg; I, 1.2 mg; Se, 0.30 mg; Ca, 8.0 g; P, 0.8 g; Sodium chloride, 5.6 g; Lysine, 0.05%.

**Table 3:** Effects of barley diets on nutrient digestibility, nitrogen balance and energy utilization of gestating sows ^1^.

Items	Basal Diet	Barley Diet ^2^										SEM	*p*-Value
		BL-1	BL-2	BL-3	BL-4	BL-5	BL-6	BL-7	BL-8	BL-9	BL-10		
BW, kg	215.78	235.13	235.13	226.83	227.07	221.80	188.13	194.30	192.17	202.20	199.53	2.91	0.33
DM intake, kg/d	2.11	2.21	2.30	2.22	2.08	2.13	1.86	1.90	1.91	1.99	1.98	0.22	0.19
Digestibility coefficient, %													
DM	88.33 ^a^	85.56 ^ab^	83.92 ^b^	84.52 ^b^	84.12 ^b^	84.38 ^b^	83.28 ^b^	84.31 ^b^	83.64 ^b^	83.97 ^b^	85.45 ^ab^	0.31	<0.01
OM	91.02 ^a^	88.50 ^a^	86.79 ^b^	87.60 ^b^	87.05 ^b^	87.31 ^b^	86.11 ^b^	87.25 ^b^	86.46 ^b^	86.84 ^b^	88.20 ^a^	0.21	<0.01
GE	87.95 ^a^	85.66 ^ab^	83.19 ^b^	84.44 ^ab^	84.78 ^ab^	84.21 ^ab^	82.52 ^b^	83.93 ^b^	83.19 ^b^	83.55 ^b^	84.88 ^a^	0.33	<0.01
CP	84.77 ^a^	83.48 ^ab^	81.76 ^abc^	82.62 ^abc^	82.55 ^abc^	82.27 ^abc^	77.55 ^c^	80.52 ^abc^	79.15 ^bc^	79.45 ^abc^	81.24 ^abc^	0.41	<0.01
NDF	52.46	51.75	52.01	44.22	43.32	49.04	43.07	38.88	43.86	42.54	52.07	1.24	0.11
ADF	55.90 ^a^	40.27 ^ab^	25.19 ^b^	27.65 ^b^	25.29 ^b^	38.21 ^ab^	27.60 ^b^	21.82 ^b^	25.88 ^b^	29.30 ^b^	40.15 ^ab^	1.92	<0.01
EE	70.33 ^a^	75.46 ^a^	71.77 ^a^	65.01 ^ab^	72.73 ^a^	53.64 ^abc^	44.16 ^bc^	41.51 ^bc^	52.54 ^abc^	46.10 ^bc^	39.60 ^c^	2.63	<0.01
Energy utilization, %													
Urinary energy, % of DE	7.12	6.90	5.53	4.97	6.56	8.14	6.78	6.61	7.74	4.96	5.56	0.51	0.92
CH_4_ energy, % of DE	0.93	0.70	0.98	0.98	0.96	0.78	0.98	0.96	0.89	1.04	0.73	0.05	0.92
ME/DE ratio	91.95	92.40	93.49	94.05	92.48	91.08	92.24	92.42	91.37	93.99	93.71	0.51	0.95
NE/ME ratio	84.79	83.36	84.23	84.80	82.72	83.23	83.91	89.92	88.82	89.63	91.26	0.76	0.13
Nitrogen balance, g/d													
Intake	48.28 ^abcd^	49.87 ^abc^	51.71 ^ab^	52.44 ^a^	47.32 ^bcde^	47.16 ^bcde^	42.81 ^e^	45.11 ^cde^	43.79 ^de^	45.47 ^cde^	44.32 ^de^	0.47	<0.01
Feces output	7.58	8.52	9.76	9.49	8.50	8.69	9.93	9.12	9.44	9.63	8.52	0.06	0.06
Urine output	2.53 ^abc^	2.84 ^abc^	3.25 ^a^	3.16 ^ab^	2.83 ^abc^	2.90 ^abc^	3.31 ^c^	3.04 ^bc^	3.15 ^bc^	3.21 ^abc^	2.84 ^abc^	0.96	<0.01
Retention	28.05	27.73	23.91	26.81	26.09	24.34	28.23	28.47	26.73	26.72	26.32	0.90	0.93

^1^ BW = body weight; DM = dry matter; GE = gross energy; CP = crude protein; NDF = neutral detergent fiber; ADF = acid detergent fiber; EE = ethyl ether extract; OM = organic matter; DE = digestible energy; ME = metabolizable energy; NE = net energy. ^2^ Diets BL-1~BL-10 were formulated by replacing corn and soybean meal in the basal diet with barley at a level of 28.9%. ^a–e^ Means within a row with different superscripts differ (*p* < 0.05).

**Table 4:** Effects of barley diets on energy balance for gestating sows ^1^.

Items	Basal Diet	Barley Diet ^2^										SEM	*p*-Value
		BL-1	BL-2	BL-3	BL-4	BL-5	BL-6	BL-7	BL-8	BL-9	BL-10		
Energy balance, kJ/kg BW^0.75^ d^−1^													
ME intake	540	528	519	530	523	512	509	518	507	524	531	4	0.59
THP	438	445	450	470	476	463	464	408	423	415	425	6	0.12
FHP	348	357	368	369	349	356	361	356	362	361	380	4	0.94
REP	73	72	55	65	72	73	79	81	76	73	72	3	0.81
REL	39 ^ab^	−35 ^c^	−25 ^ab^	−33 ^c^	−30 ^c^	−44 ^c^	−11 ^ab^	57 ^a^	35 ^ab^	37 ^ab^	56 ^a^	7	<0.01
RE	111 ^abc^	37 ^d^	30 ^d^	32 ^d^	42 ^bc^	29 ^d^	68 ^abc^	138 ^a^	111 ^abc^	109 ^abc^	128 ^ab^	7	<0.01
RQ													
Fed state	0.99 ^ab^	0.93 ^b^	0.93 ^b^	0.93 ^b^	1.00 ^a^	1.00 ^a^	0.97 ^ab^	0.98 ^ab^	0.96 ^ab^	1.00 ^a^	0.99 ^ab^	0.01	<0.05
Fasted state	0.81 ^ab^	0.76 ^b^	0.77 ^b^	0.76 ^b^	0.83 ^a^	0.83 ^a^	0.80 ^ab^	0.78 ^ab^	0.78 ^ab^	0.81 ^ab^	0.81 ^ab^	0.01	<0.05
Energy values, MJ/kg DM													
DE	15.62 ^ab^	15.53 ^abc^	14.50 ^d^	14.84 ^cd^	15.88 ^ab^	15.13 ^bcd^	15.00 ^bcd^	15.32 ^abc^	15.01 ^bcd^	15.00 ^bcd^	15.18 ^cd^	0.06	<0.01
ME	14.37	14.35	13.56	13.96	14.69	13.78	13.85	14.16	13.71	14.10	14.23	0.10	0.44
NE	12.23	11.98	11.42	11.84	12.16	11.50	11.65	12.75	12.24	12.65	13.02	0.16	0.59

^1^ BW = body weight; THP = total heat production; FHP = fasting heat production; REP = energy retention as protein; REL = energy retention as lipid; RE = energy retention; RQ = respiratory quotient; DE = digestible energy; ME = metabolizable energy; NE = net energy. ^2^ Diets BL-1~BL-10 were formulated by replacing corn and soybean meal in the basal diet with barley at a level of 28.9%. ^a–d^ Means within a row with different superscripts differ (*p* < 0.05).

**Table 5:** Digestibility and energy values of barley ^1^.

Items	Barley										SEM	*p*-Value
	Barley-1	Barley-2	Barley-3	Barley-4	Barley-5	Barley-6	Barley-7	Barley-8	Barley-9	Barley-10		
Digestibility coefficient, %												
DM	73.58	75.65	74.27	75.15	71.35	73.43	73.92	71.60	72.76	77.88	0.95	0.93
OM	90.82	90.75	90.78	90.77	90.78	90.98	91.02	90.99	91.00	91.05	0.17	0.13
GE	80.50	71.91	76.16	77.17	75.62	72.37	74.29	72.20	73.44	77.77	0.93	0.53
CP	79.28	70.48	73.79	75.22	74.90	52.68	62.33	65.52	66.91	71.89	1.60	0.11
NDF	46.19	47.09	41.77	34.77	51.21	36.31	37.56	39.42	38.07	71.63	3.54	0.49
ADF	8.25 ^ab^	6.99 ^b^	4.67 ^c^	10.88 ^a^	7.92 ^b^	5.47 ^bc^	4.02 ^c^	6.47 ^b^	5.63 ^bc^	4.91 ^c^	0.67	<0.01
Energy values, MJ/kg DM												
DE	15.62 ^b^	12.08 ^b^	13.25 ^b^	16.83 ^a^	17.28 ^a^	14.66 ^b^	15.78 ^a^	14.70 ^b^	14.68 ^b^	15.29 ^b^	0.25	<0.01
ME	14.28	11.74	13.03	15.39	16.12	13.13	14.14	12.66	13.94	14.36	0.36	0.22
NE	9.22	7.85	8.88	9.66	11.61	8.49	11.18	9.95	10.95	11.85	0.43	0.23
Energy utilization, %												
ME/DE	91.45	97.12	98.59	91.09	92.88	88.13	89.41	86.75	95.09	93.55	1.75	0.91
NE/ME	63.43	67.21	68.11	62.23	69.08	62.25	78.83	74.27	77.93	80.81	2.09	0.33

^1^ DM = dry matter; GE = gross energy; CP = crude protein; NDF = neutral detergent fiber; ADF = acid detergent fiber; OM = Organic matter; DE = digestible energy; ME = metabolizable energy; NE = net energy; Barley-1 to Barley-10 are the 10 types of barley used in the experiment. ^a–c^ Means within a row with different superscripts differ (*p* < 0.05).

**Table 6:** Correlation analysis of barley energy value and chemical composition ^1^.

Items	CP	NDF	ADF	Hemicellulose	Ash	EE	Starch	IDF	SDF	TDF	GE	DE	ME	NE
CP	1													
NDF	−0.07	1												
ADF	0.46	0.42	1											
Hemicellulose	−0.12	0.99 **	0.32	1										
Ash	0.60 *	0.35	0.53	0.30	1									
EE	0.46	−0.18	−0.04	−0.18	−0.19	1								
Starch	−0.10	−0.59 *	−0.38	−0.57	−0.67 *	0.19	1							
IDF	0.61 *	0.41	0.94 **	0.32	0.59 *	0.02	−0.32	1						
SDF	−0.33	0.67	0.39	0.66 *	0.37	−0.50	−0.47	0.36	1					
TDF	0.45	0.54	0.92 **	0.45	0.62 *	−0.12	−0.40	0.97 **	0.57 *	1				
GE	0.76 *	−0.01	0.37	−0.05	0.65 *	0.16	−0.48	0.40	−0.37	0.26	1			
DE	−0.66 *	0.03	−0.56 *	0.10	−0.18	−0.55	0.05	−0.46	0.45	−0.29	−0.61 *	1		
ME	−0.57	−0.07	−0.49	−0.02	0.01	−0.65 *	0.01	−0.44	0.44	−0.27	−0.47	0.93 **	1	
NE	−0.10	0.06	−0.47	0.12	0.17	−0.14	0.02	−0.30	0.33	−0.17	−0.33	0.65 *	0.67 *	1

^1^ CP = crude protein; NDF = neutral detergent fiber; ADF = acid detergent fiber; EE = ethyl ether extract; IDF = insoluble dietary fiber; SDF = soluble dietary fiber; TDF = total dietary fiber; GE = gross energy; DE = digestible energy; ME = metabolizable energy; NE = net energy. * *p* < 0.05, ** *p* < 0.01.

**Table 7:** Net energy prediction equation for barley fed to gestating sows.

No.	Barley Net Energy Prediction Equation (*n* = 10) ^1^	R^2^	RMSE ^2^	*p*-Value
1	NE = 4.35 − 3.92 ADF + 1.24 TDF	0.66	0.90	0.01
2	NE = 10.21 + 0.93 ME + 0.51 CP	0.62	0.93	0.03

^1^ The value of the energy and chemical composition in the equations was expressed on a dry matter basis. ^2^ RMSE: root mean square error is a measure of precision.

## Data Availability

The original contributions presented in this study are included in the article. Further inquiries can be directed to the corresponding author.
